# Using Bronson Equation to Accurately Predict the Dog Brain Weight Based on Body Weight Parameter

**DOI:** 10.3390/vetsci3040036

**Published:** 2016-12-04

**Authors:** L. Miguel Carreira

**Affiliations:** 1 Faculty of Veterinary Medicine, University of Lisbon (FMV/ULisboa), Av. da Universidade Técnica de Lisboa, Polo Universitário Alto da Ajuda, Lisbon 1300-477, Portugal; miguelcarreira@fmv.ulisboa.pt; 2 Centre for Interdisciplinary Research in Animal Health (CIISA), FMV/ULisboa, Av. da Universidade Técnica de Lisboa, Polo Universitário Alto da Ajuda, Lisbon 1300-477, Portugal; 3 Anjos of Assis Veterinary Medicine Centre (CMVAA), Rua Dª. Francisca da Azambuja Nº 9–9A, Barreiro 2830-077, Portugal; 4 Private Practice in Human Dentistry, Oral Medicine and Surgery Clinic, Lisbon 1300-477, Portugal

**Keywords:** dog, brain, brachycephalic, dolichocephalic, mesaticephalic, body weight

## Abstract

The study used 69 brains (*n* = 69) from adult dog cadavers, divided by their skull type into three groups, brachi (B), dolicho (D) and mesaticephalic (M) (*n* = 23 each), and aimed: (1) to determine whether the Bronson equation may be applied, without reservation, to estimate brain weight (BW) in brachy (B), dolicho (D), and mesaticephalic (M) dog breeds; and (2) to evaluate which breeds are more closely related to each other in an evolutionary scenario. All subjects were identified by sex, age, breed, and body weight (bw). An oscillating saw was used for a circumferential craniotomy to open the skulls; the brains were removed and weighed using a digital scale. For statistical analysis, *p-*values < 0.05 were considered significant. The work demonstrated a strong relationship between the observed and predicted BW by using the Bronson equation. It was possible to hypothesize that groups B and D present a greater encephalization level than M breeds, that B and D dog breeds are more closely related to each other than to M, and from the three groups, the D individuals presented the highest brain mass mean.

## 1. Introduction

Body weight influences almost every aspect of organism biology, including brain mass [1]. Variations in brain mass occur in proportion to the increase of body mass of the animal (allometric scaling); by increasing body size and weight, an increase of peripheral receptor conduction will promote a rise in the corresponding representation fields in the cerebral cortex (both motor and somatosensory cortices) [2,3,4]. Several studies demonstrated a strong positive correlation between brain weight (BW) and body weight (bw) in general and specifically in dogs [5,6,7,8,9,10,11,12]. The brain is at adult size when body growth is only 40 percent complete [13,14,15,16,17]. Bronson developed a mathematical equation to explain the encephalic allometry, demonstrating that the mean BW of dogs is related to mean bw and therefore BW could be calculated without previous models’ inconsistencies The evolution of diversity in brain size [3,18,19,20,21,22,23,24]. According to Bronson’s dog equation, average BW (y) can be expressed by the following allometric function: *Y* = 0.39*X*^0.27^, where 0.39 is the integration constant, *X* is the bw mean, and 0.27 is the allometric exponent or the potency associated with the intra-species ratio of BW and bw means [3,18,25,26,27]. This function may serve as a baseline for measuring increases or decreases in encephalization during species evolution [3]. Encephalization level (El) as a measure of relative brain size—defined as the ratio between the actual and the predicted brain mass for an individual El—is also used as a rough estimate of the intelligence of the animal and is useful for comparison within species or between fairly closely related species [28]. Since the allometric coefficient often exhibits intraspecific variation, which can be heritable, some theories assume that greater BW differences are a result of selection for differences in bw, registered among more distantly than closely related species [5,15]. The present study was developed in dog specimens and aimed: (1) to determine whether the Bronson equation may be applied, without reservation, to estimate BW in all the brachy (B), dolicho (D), and mesaticephalic (M) dog breeds; and (2) to evaluate which breeds are more closely related to each other in an evolutionary scenario.

## 2. Materials and Methods

The study used 69 brains (*n* = 69) from adult dog cadavers, divided by their skull type into three groups, B, D, and M (*n* = 23 each), obtained at the teaching hospital of Faculty of Veterinary Medicine, University of Lisbon (FMV-ULisboa), Portugal, and Anjos of Assis Veterinary Medicine Centre (CMVAA), Barreiro, Portugal, after verification of death by a veterinary surgeon, authorization by the dog owners with signed consent forms, and approval by the ethical committee. All subjects were identified by sex, age, breed, and bw. An oscillating saw was used for a circumferential craniotomy to open the skulls; the brains were removed and weighed using a digital scale. The measurements were recorded onto recording forms, and the data were put into an SPSS^®^ database (IBM, Armonk, North Castle, NY, USA). The Kolgomorov-Smirnov test (KS) was used to test for normality and ANOVA was used to test if BW differed among B, D, and M breeds. To test for pair-wise differences between groups, we used a post hoc Bonferroni correction. A *p*-value < 0.05 was considered statistically significant.

## 3. Results

Summary statistics for all considered variables (age, bw, and BW) are listed in Table 1. From all the evaluated parameters, only the BW of the brachycephalic group did not show data normality. The Bronson equation was used to calculate the expected BW from each specimen. Table 2 presents the one-way ANOVA results that tested if BW differed among B, D, and M breeds, and the post hoc Bonferroni correction to test for significant pair-wise differences between groups. Differences were registered for the bw and BW parameters. Statistically significant differences were registered for bw between the pair groups B-D (*p* < 0.00) and D-M (*p* < 0.00), and for BW only between the pair groups D-M (*p* < 0.00).

## 4. Discussion

According to the results, BW mean differed little, with no statistical significant differences, between the three groups considered; however, from all the groups, the M group presented a greater mean age (9.5 years), followed by group D (8.7 years), and then B (8.0 years). This is a very important parameter, since aging is associated with decreased brain volume (about 0.29% per year) and weight (about 5%–10% over the lifetime), due to atrophy of the gray and white matters, enhanced by multiple factors [17,29,30,31,32]. Aged dog brains show increased cortical atrophy, ventricular dilation, decreased total brain volume, and decreased frontal lobe volume, similar to humans [29,32]. In previous studies, age-related reduction in gray matter volume was observed bilaterally in the frontal gyrus, orbitalis gyrus, ectosylvius hyrus, olfactory bulb, superior olivaris nucleus in brainstem, and unilaterally in the posreal gyrus, sylvium gyrus, suprasylvian gyrus, cerebellum, and brainstem nuclei. Variations related to white matter loss were largely bilateral and included the internal capsula, tracts of anterior cingulate, and the alveus of the hypocampus [17,29,32]. Additionally, high body mass index, biochemical changes (in particular, those related to dopamine loss), and the loss of neurons and myelinated axons in different brain parenchyma regions decrease the brain mass with age promoting an enlargement of the ventricular system and the grooves on the brain surface [17,30,31,32,33,34]. In accordance with Schmidt et al. [35], the Bronson equation may serve as a baseline for measuring the El of species during their evolution. According to Kruska [5] and Pagel and Harvey [15], greater BW differences are registered among distantly related species as a result of selection for differences in bw. The study results showed that the biggest differences were registered between the pair-wise groups M-B and M-D; thus we can conclude that B and D dog breeds are more closely related to each other than to M. This is supported by the fact that the B and D breeds were the result of natural and artificial selection over the M breeds, which are most closely related to the gray wolf—*Canis lupus*—and from which they were domesticated [36]. This selection acted directly on the brain itself, and also depended on the selective forces involved, promoting the decreasing size of one part of the brain in order to increase the size of another [35,37]. This is the result of simultaneous isocortical neurogenesis onset and terminal neurogenesis that varies substantially between the rostral and caudal poles in some species, allowing for disproportionate expansion of some encephalic cortical regions relative to others [38,39,40,41,42,43]. With the Bronson equation, it was possible to verify that D and B specimens presented the best ratio between the registered and the predicted BW, and according the study results, we found that D has the highest mean brain mass, heavier than the B and the M specimens.

## 5. Conclusions

In conclusion, the work demonstrated a strong relationship between the observed and predicted BW by using the Bronson equation. It was possible to hypothesize that groups B and D present a greater El than M breeds, and from the three groups, the D individuals presented the highest brain mass mean.

## Figures and Tables

**Table 1:** Descriptive statistics with mean, standard deviation, minimum and maximum values, sigma and *t*, regarding the parameters of age, living weight, brain weight, and breeds in brachy (B), dolicho (D) and mesaticephalic (M) dogs.

Group	Parameter	*n*		$\bar{x}$ ± SD	95%CI		σ for *p* > 0.05	*t*
					Min (mm)	Max (mm)		
**B**	**age**	23		8.00 ± 1.65 ^¥^	4.00	10.00	σ > 0.10	20.13
	**bw**	23		15.79 ± 6.46 ^§^	4.70	26.10	σ > 0.10	11.71
	**BW**	23		84.91 ± 31.29 *	26.62	147.27	σ = 0.08	13.02
	**Breed**	23	8	French Bulldog				
			7	Boxer				
			4	Pekingese				
			2	Pug Carlin				
			2	Shitzu				
**D**	**age**	23		8.70 ± 2.40 ^¥^	5.00	14.00	σ > 0.10	17.46
	**bw**	23		23.63 ± 2.59 ^§^	18.70	29.39	σ > 0.10	37.79
	**BW**	23		92.50 ± 8.60 *	71.60	102.10	σ = 0.00	50.60
	**Breed**	23	11	Doberman Pinsher				
			5	Rough Collie				
			4	Whippet				
			3	Miniature Bull Terrier				
**M**	**age**	23		9.50 ± 2.50 ^¥^	5.00	15.00	σ = 0.02	17.70
	**bw**	23		13.80 ± 7.20 ^§^	7.30	31.50	σ < 0.01	8.74
	**BW**	23		69.90 ± 28.70 *	35.70	143.0	σ = 0.04	11.68
	**Breed**	23	7	Beagle				
			6	Golden Retriever				
			5	Yorkshire Terrier				
			3	Border Collie				
			2	Dalmatian				

Body weight (bw); brain weight (BW), minimum (min); maximum (max); confidence interval (CI). Measurements are in millimetres*.* **^¥^** Years, **^§^** kilograms, * grams.

**Table 2:** One-way ANOVA and post hoc Bonferroni corrected tests for differences in age, living weight, brain weight, maximum width, length and height between the brachy (B), dolicho (D) and mesaticephalic (M) dogs. The comparison between groups was made with an *n* = 46 (23 specimens within each group). Also, the comparison between observed and expected brain weight values obtained by using the Bronson equation in brachy (B), dolicho (D) and mesaticephalic (M) dogs. The correlation coefficient (Pearson’s product moment correlation) between both values in the three considered groups. The difference was significant at *p*-values < 0.05.

Sample Characteristics													
Parameter	Type of Test		Differences Between Groups		*n*	DM (I–J)	EP	SS	MS	F	Sig.	CI 95%	
												Min	Max
Age	ANOVA		-		69	-	-	28.17	14.08	2.77	0.06	-	-
	Bonferroni		B	D	46	–0.78	1.17	-	-	-	1.00	–2.41	0.85
			B	M	46	–1.56	2.35	-	-	-	0.09	–3.19	0.06
			D	M	46	–0.78	1.17	-	-	-	0.39	–2.41	0.85
bw	ANOVA		-		69	-	-	3303	447.7	12.27	0.00	-	-
	Bonferroni		B	D	46	–6.62 *	3.72	-	-	-	0.00	–11.00	2.25
			B	M	46	1.73	0.97	-	-	-	1.00	–2.64	6.10
			D	M	46	8.36 *	4.69	-	-	-	0.00	–3.98	12.73
BW	ANOVA		-		69	-	-	4739	3026	4.83	0.01	-	-
	Bonferroni		B	D	46	–7.62	1.03	-	-	-	1.00	–25.75	10.50
			B	M	46	14.92	2.02	-	-	-	>0.05	–3.20	33.06
			D	M	46	22.55 *	3.05	-	-	-	0.00	4.41	40.68
Brain Weight													
Group		n		Observed BW (g)			Expected BW (g)			*p*	r	χ^2^ for χ^2c^ > 5.99	
				$\bar{x}$	min	max	$\bar{x}$	min	max				
B		23		84.91	53.60	116.20	82.20	71.30	90.10	0.68	0.99	2.47	
D		23		92.50	83.90	101.10	91.30	88.50	94.00	0.35	0.81	1.28	
M		23		69.90	41.20	98.60	79.20	64.60	88.80	0.17	0.96	5.85	

Body weight (bw); brain weight (BW); Mean Difference (I–J); confidence interval (CI); minimum (min); maximum (max); Pearson correlation coefficient (r); Chi-square (χ^c^); Chi-square critical (χ^2c^); * statistically significant.
